# Investigation of PD-L1 Expression in Various Non-Hodgkin's B-cell Lymphomas

**DOI:** 10.30699/ijp.2025.2051609.3406

**Published:** 2025-08-15

**Authors:** Pardis Nematollahi, Shiva Radfar

**Affiliations:** 1 Department of Pathology, School of Medicine, Cancer Prevention Research Center, Seyyed Al-Shohada Hospital, Isfahan University of Medical Sciences, Isfahan, Iran; 2 Department of Pathology, School of Medicine, Isfahan University of Medical Sciences, Isfahan, Iran

**Keywords:** PD-L1; Non-Hodgkin’s lymphoma; immunophenotype; immunohistochemistry; DLBCL; cancer immunotherapy

## Abstract

**Background & Objective:**

Programmed death ligand-1 (PD-L1) plays a critical role in tumor immune evasion, particularly in Non-Hodgkin’s B-cell lymphomas (NHL). This study aimed to evaluate PD-L1 expression across various NHL immunophenotypes and assess its correlation with clinical and demographic parameters.

**Methods:**

In this cross-sectional, descriptive-analytical study, 71 formalin-fixed, paraffin-embedded tissue blocks diagnosed with Non-Hodgkin’s B-cell lymphoma were retrieved from the pathology archives of Alzahra Hospital and Seyed-al Shohada (Omid) Hospital in Isfahan (2019–2020). PD-L1 expression was assessed immunohistochemically using tumor proportion score (TPS), combined proportion score (CPS), and immune cell (IC) score. Statistical analysis was performed using the Mann-Whitney U and Kruskal-Wallis tests, with p < 0.05 considered statistically significant.

**Results:**

Among the 71 patients, 67.6% were male, with a mean age of 52.61 ± 18.43 years. Diffuse large B-cell lymphoma (DLBCL) was the most common subtype, accounting for 52.1% of cases. PD-L1 expression was significantly higher in females (TPS: 18.13 ± 9.73; CPS: 28.22 ± 13.31) compared to males (TPS: 4.42 ± 3.56; CPS: 12.08 ± 10.14) (p = 0.040, p = 0.022). No significant differences in PD-L1 expression were observed across age groups. DLBCL demonstrated significantly higher IC and CPS values compared to other subtypes (p < 0.05), while plasmacytoma and Burkitt lymphoma exhibited the lowest scores (e.g., immune score: 1.11 ± 0.11 for plasmacytoma). No statistically significant differences in TPS were noted among the different immunophenotypes (p = 0.119).

**Conclusion:**

Elevated PD-L1 expression, particularly in immune cell scores, suggests potential utility in PD-1/PD-L1–targeted therapies for NHL. However, the prognostic significance of PD-L1 remains inconclusive, highlighting the need for further investigation into its role as a predictive biomarker in the clinical management of Non-Hodgkin’s B-cell lymphomas.

## Introduction

B-cell lymphomas are a heterogeneous group of neoplasms arising from the clonal expansion and proliferation of either germinal center or non-germinal center B cells, and they exhibit considerable genetic and biological diversity (1). According to the American Cancer Society, B-cell lymphomas constitute approximately 85% of all non-Hodgkin lymphomas (NHL). Based on biological behavior, B-cell lymphomas are classified into high-grade and low-grade subtypes. Among them, diffuse large B-cell lymphoma (DLBCL) is the most common (30%), followed by follicular lymphoma (20%). Some B-cell lymphomas exhibit resistance to conventional therapies such as chemotherapy and radiotherapy and may benefit from targeted immunotherapeutic approaches (2).

Immune checkpoint inhibitors are among the most promising immunotherapies and include molecules targeting the programmed death-1 (PD-1) receptor and its ligand, programmed death ligand-1 (PD-L1). PD-L1 is a co-inhibitory molecule expressed on various immune cells, including CD4+ and CD8+ T cells. When PD-1 binds to its ligands—PD-L1 or PD-L2, which are expressed on tumor cells and antigen-presenting cells—it suppresses T-cell activation by reducing interleukin-2 (IL-2) production and T-cell proliferation. This immune evasion mechanism promotes tumor survival and progression (3). The PD-1/PD-L1 axis plays a critical role in both autoimmune disorders and neoplastic diseases. Although several studies have highlighted the clinical relevance of PD-L1 expression in guiding therapeutic decisions, its prognostic value remains controversial (4–8).

PD-L1 expression can be detected by immunohistochemistry (IHC) in tissue sections (9) and by enzyme-linked immunosorbent assay (ELISA) in blood samples (10). Under physiological conditions, PD-L1 expression is limited to specific tissues such as the placenta, heart, lungs, and skeletal muscle, and to select immune cell populations including hematopoietic cells, antigen-presenting cells (APCs), and activated T cells (4, 5, 11, 12). However, its expression is markedly upregulated in various malignancies, including carcinomas, melanomas, and several lymphoid neoplasms (6, 13–15). Elevated PD-L1 expression has been documented in hematolymphoid malignancies such as angioimmunoblastic T-cell lymphoma, follicular lymphoma, DLBCL, primary mediastinal B-cell lymphoma, and classical Hodgkin lymphoma—particularly the nodular sclerosis subtype (16, 17). Although PD-L1 overexpression has been associated with reduced survival in numerous solid and hematologic tumors (18, 19), its precise prognostic role in lymphomas remains under debate.

Beyond expression levels, the pattern of PD-L1 expression in tumors is variable. In some malignancies, heterogeneous PD-L1 expression is observed and is thought to result from the activity of tumor-infiltrating lymphocytes (TILs), particularly via the secretion of interferons (20–22). In contrast, homogeneous PD-L1 expression has been reported in tumors such as classical Hodgkin lymphoma, particularly in Reed–Sternberg cells, and is believed to be driven by intrinsic genetic or epigenetic mechanisms (23, 24).

Recent advancements in genomic technologies have significantly enhanced our understanding of the molecular mechanisms underlying lymphoid malignancies. In parallel, the field of immunotherapy has provided new avenues for the treatment of these diseases. Among these approaches are monoclonal antibodies, anti-idiotype vaccines, and immune checkpoint inhibitors. However, the successful application of immunotherapy requires confirmation of the presence of the relevant molecular targets in the tumor. One such promising target is the PD-1/PD-L1 axis.

Therefore, this study aimed to evaluate the expression of PD-L1 in various immunophenotypes of B-cell non-Hodgkin lymphomas and investigate its correlation with demographic and clinical features.

## Materials and Methods

This cross-sectional, descriptive-analytical study included paraffin-embedded tissue blocks with a confirmed diagnosis of Non-Hodgkin’s B-cell lymphoma (NHL), obtained from the pathology archives of Alzahra Hospital and Seyed-al Shohada (Omid) Hospital, affiliated with Isfahan University of Medical Sciences, Isfahan, Iran, between 2019 and 2020.

Due to the limited number of eligible cases, a census sampling method was used, including all archived samples that met the inclusion criteria. Initial estimations suggested that approximately 80 cases would be eligible.

Ethical approval was obtained from the Ethics Committee of Isfahan University of Medical Sciences prior to sample collection. Inclusion criteria were: 1) a final, definitive diagnosis of Non-Hodgkin’s B-cell lymphoma based on WHO classification; 2) availability of paraffin blocks with sufficient tumor tissue; and 3) access to complete demographic data. Exclusion criteria included: 1) sample contamination and 2) history of prior radiotherapy or chemotherapy. In cases where multiple blocks were available for the same tumor, the block with the highest tumor cellularity was selected, due to the known heterogeneity of PD-L1 expression.

Immunohistochemistry (IHC)

Immunohistochemical analysis was performed using a monoclonal anti–PD-L1 antibody (clone CAL10, Master Diagnóstica, Spain), following antigen retrieval as per the manufacturer’s instructions. Placenta tissue was used as the positive control for PD-L1. PD-L1 expression was evaluated on tumor cells, tumor-infiltrating lymphocytes (TILs), and tumor-associated macrophages (TAMs). All IHC slides were reviewed semi-quantitatively under a light microscope by two independent pathologists blinded to clinical data, using a multi-headed microscope to ensure consensus.

PD-L1 Scoring Criteria:

1. **Tumor Proportion Score (TPS):** The percentage of PD-L1–positive tumor cells among all viable tumor cells.
2. **Combined Proportion Score (CPS):** The ratio of all PD-L1–positive tumor and immune cells (lymphocytes and macrophages) to the total number of viable tumor cells, multiplied by 100.
3. **Immune Cell Score (IC):** The percentage of the tumor and peritumoral area occupied by PD-L1–positive immune cells (14).

### Data Analysis

Data were analyzed using SPSS software (version 26). Results are presented as mean ± standard deviation or frequency (percentage). The Kolmogorov-Smirnov test was used to assess normality, and non-parametric tests were applied due to non-normal distribution. The Mann–Whitney U test was used to compare PD-L1 expression levels by gender and age group, as well as for pairwise comparisons of TPS and IC scores. The Kruskal–Wallis** test** was employed to compare PD-L1 expression across different immunophenotypes. A p-value < 0.05 was considered statistically significant for all analyses.

**Table 1:** Baseline characteristics and tumor immunophenotype in patients

Characteristics		N(%) or mean ± SD
Sex	Male	48(67.6%)
	Female	23(32.4%)
Age; year		52.61±18.43
Tumor Immunophenotype	DLBCL	37(52.1%)
	FL	10(14.1%)
	MCL	3(4.2%)
	MZL	2(2.8%)
	SLL	5(7.0%)
	Plasmacytoma	9(12.7%)
	BL	5(7.0%)

DLBC: diffuse large B-cell, FL: Follicular lymphoma, MCL: Mantle cell lymphoma, MZL: Marginal zone lymphoma, SLL: Small lymphocytic leukaemia, BL: Burkitt lymphoma

## Results

This study was conducted on 71 paraffin blocks with a final diagnosis of Non-Hodgkin’s B-cell lymphoma, belonging to 48 males (67.6%) and 23 females (32.4%) with a mean age of 52.61±18.43 years. The immunophenotype of these patients showed the highest percentage (52.1%) in the DLBCL type and the lowest percentage (2.8%) in the MZL type (Table 1).

Analysis of PD-L1 expression percentage by gender and age revealed that in men, the PD-L1 expression percentage in tumor proportion score and combined proportion score was significantly lower than in women, with the mean of 4.42±3.56 and 12.08±10.14, respectively, compared to 18.13±9.73 and 28.22±13.31 in women (P value=0.040, 0.022). However, there was no significant difference in PD-L1 expression in immune score between the two genders (P value>0.05). Furthermore, analysis of PD-L1 expression in each gender (male and female) and in each age group (< or ≥ 50 years) showed no significant difference in the mean of PD-L1 expression in tumor proportion score and immune score (P value>0.05) (Table 2).

**Table 2:** Determination and comparison of mean PDL-1 expression percentage based on gender and age of the patients.

Characteristics	PDL-1 expression			P1
	Tumor proportion score	Immunescore	Combined proportion score	
Sex				
Male	4.42±3.56	6.56±5.68	12.08±10.14	0.116
Female	18.13±9.73	10.22±9.31	28.22±13.31	0.245
P_2_	0.040	0.187	0.022	
Age; year				
< 50 year	13.67±26.56	8.47±10.59	23.97±29.25	0.323
≥ 50 year	5.34±9.11	7.22±6.67	12.44±12.80	0.290
P_2_	0.830	0.911	0.340	

DLBC: diffuse large B-cell, FL: Follicular lymphoma, MCL: Mantle cell lymphoma, MZL: Marginal zone lymphoma, SLL: Small lymphocytic leukaemia, BL: Burkitt lymphoma

P1: The significance level obtained from the Mann-Whitney test for comparing the mean of PDL-1 expression between Tumor proportion score and Immuno score. P2: The significance level obtained from the Mann-Whitney test for comparing the mean percentage of PDL-1 expression by gender or age group.

Conversely, the expression of PDL-1 in tumor proportion score did not show a significant difference between different immunophenotypes (P value=0.119); yet, the mean immune score and combined poroportion score differed significantly between different immunophenotypes (P value<0.05). Notably, the mean expression of PDL-1 based on immune score and combined proportion score was significantly higher in the DLBCL immunophenotype compared to other immunophenotypes, with the lowest mean observed in the plasmacytoma and BL. Furthermore, it was found that in some immunophenotypes such as MCL, MZL, DLBCL, and SLL, the mean expression of PDL-1 based on tumor proportion score and immune score did not differ significantly (P value>0.05). However, in other immunophenotypes (including FL, Plasmacytoma, and BL), the mean expression of PDL-1 in immune score was significantly higher than tumor proportion score (P value<0.05) (Table 3).

**Table 3:** Determination and comparison of the mean percentage of PD-L1 expression according to the type of tumor immunophenotype

Characteristics	PD-L1 expression			P1
	Tumor proportion score	Immunescore	Combined proportion score	
Tumor Immunophenotype				
DLBCL	15.57±24.20	9.86±1.65	26.89±4.29	0.073
FL	3.70±1.90	9.50±1.91	13.00±2.91	0.021
MCL	2.33±1.33	10.00±2.89	12.00±4.16	0.073
MZL	1.00±0.50	12.00±3.00	12.50±3.54	0.067
SLL	1.20±1.30	3.80±2.08	5.00±2.59	0.264
Plasmacytoma	0±0	1.11±0.11	1.11±0.11	<0.001
BL	0.20±0.20	1.40±0.89	1.60±0.89	0.037
P_2_	0.119	0.039	0.006	

DLBC: diffuse large B-cell, FL: Follicular lymphoma, MCL: Mantle cell lymphoma, MZL: Marginal zone lymphoma, SLL: Small lymphocytic leukaemia, BL: Burkitt lymphoma

P1: The significance level obtained from the Mann-Whitney test for comparing the mean expression of PDL-1 between Tumor proportion score and Immuno score.

P2: The significance level obtained from the Mann-Whitney test for comparing the mean percentage of PDL-1 expression by immunophenotype.

## Discussion

Lymphomas utilize various immune evasion mechanisms, and the PD-1/PD-L1 signaling axis plays a critical role by inhibiting lymphocyte activation and promoting tumor immune escape (25,26). PD-L1 expression has been widely reported on both tumor cells and tumor-infiltrating immune cells (27). However, its presence on circulating lymphocytes and its potential role in systemic immunosuppression remain poorly understood.

In the present study, we evaluated PD-L1 expression in patients with B-cell non-Hodgkin lymphoma (NHL). Although the mean PD-L1 expression was numerically higher in the tumor proportion score (TPS) than in the immune cell score (IC), this difference was not statistically significant. Notably, PD-L1 expression in TPS was significantly higher in females compared to males, while IC scores showed no significant gender difference. This may suggest that tumor cell–associated PD-L1 expression is influenced by gender-specific biological or immunologic factors, whereas immune cell expression is less variable across sexes.

Age did not significantly correlate with PD-L1 expression across any scoring method, indicating that PD-L1-targeted therapies may be applicable across different age groups. When comparing PD-L1 expression by immunophenotype, no statistically significant difference was observed in TPS (p = 0.119); however, IC scores revealed a significant variation (p < 0.05), with diffuse large B-cell lymphoma (DLBCL) showing the highest PD-L1 expression, and plasmacytoma and Burkitt lymphoma (BL) the lowest. Interestingly, in follicular lymphoma (FL), plasmacytoma, and BL, IC scores were significantly higher than TPS, suggesting greater PD-L1 expression in the tumor microenvironment than in tumor cells themselves.

Several studies have investigated the PD-L1 pathway in DLBCL and other lymphomas. Ibrahim et al. found no significant association between PD-L1 expression and factors such as age, gender, serum LDH, or B symptoms, but did find a significant correlation with high International Prognostic Index (IPI) scores and poor prognosis (28). These findings are consistent with Zhao et al., who conducted a meta-analysis of nine studies (including five on DLBCL) and reported no association between PD-L1 and Ann Arbor staging. However, they highlighted limitations such as variability in antibody clones, inconsistent cut-off values, and publication bias (29). Similarly, Zeng et al., in a meta-analysis of 12 studies (six involving DLBCL), reported that PD-L1 overexpression was associated with B symptoms, advanced stage (Ann Arbor III/IV), higher IPI scores, and worse prognosis (30). Qiu et al. also reported that high PD-L1 expression was associated with significantly shorter progression-free and overall survival, particularly when PD-L1 positivity was ≥30% (31).

Recent research indicates that both the extent and intensity of PD-L1 expression may affect the efficacy of PD-1/PD-L1 inhibitors (32). This underscores the importance of standardized and validated scoring systems. New technologies such as multiplex immunohistochemistry and digital image analysis may provide real-time, high-resolution evaluation of PD-L1 expression and its spatial relationship with immune cells.

Additionally, the interplay between PD-L1 expression and tumor-infiltrating lymphocytes (TILs), particularly CD8+ T cells, adds complexity. In some tumors, PD-L1 expression may be induced by interferon-γ released by activated TILs, reflecting an adaptive immune resistance mechanism (33). This highlights the potential value of combining PD-L1 and TIL assessments to refine prognostication and treatment selection.

Therapeutically, PD-L1 expression may inform the use of immune checkpoint inhibitors, including in combination with emerging treatments such as CAR T-cell therapy or bispecific antibodies (34). Understanding how PD-L1 expression affects treatment response could help personalize immunotherapy regimens.

Despite its promise, several challenges remain. These include technical variability in immunohistochemistry (IHC) assays, inconsistencies in tissue handling and fixation, and lack of consensus on positivity thresholds (35). Furthermore, in primary DLBCL and central nervous system (CNS) lymphomas, PD-L1 plays a key role in fostering immune escape and disease progression (36). Addressing these limitations through standardized protocols will be crucial for integrating PD-L1 testing into routine clinical practice.

Our findings demonstrated significantly higher TPS and CPS values in female patients and elevated IC scores in DLBCL compared to other subtypes. These observations support PD-L1’s utility as a therapeutic biomarker, especially in DLBCL. In contrast, plasmacytoma and BL showed minimal expression, reflecting their distinct tumor biology and potential resistance to PD-1/PD-L1 blockade.

## Conclusion

This study highlights the variable expression of PD-L1 in B-cell NHL, with significantly higher expression in females and in DLBCL cases. PD-L1 levels were not affected by age, suggesting broad applicability of checkpoint inhibitors across age groups. The marked differences in immune scores across immunophenotypes, particularly the high levels in DLBCL, point to its potential as a predictive biomarker and immunotherapeutic target.

However, translating these findings into clinical application requires standardization of PD-L1 testing protocols and scoring systems. Future multicenter studies with larger cohorts and uniform methodologies are essential to validate these observations and enhance the role of PD-L1 in personalizing treatment strategies for patients with NHL.

## Data Availability

The data that support the findings of this study are available from the corresponding author upon reasonable request.
